# Analyzing differences between parent- and self-report measures with a latent space approach

**DOI:** 10.1371/journal.pone.0269376

**Published:** 2022-06-29

**Authors:** Dongyoung Go, Minjeong Jeon, Saebyul Lee, Ick Hoon Jin, Hae-Jeong Park

**Affiliations:** 1 Department of Applied Statistics, Yonsei University, Seoul, Republic of Korea; 2 Department of Statistics and Data Science, Yonsei University, Seoul, Republic of Korea; 3 School of Education and Information Studies, University of California, California, Los Angeles, United States of America; 4 Graduate School of Medical Science, Brain Korea 21 Project, Yonsei University College of Medicine, Seoul, Republic of Korea; 5 Department of Nuclear Medicine, Department of Psychiatry, Yonsei University College of Medicine, Seoul, Republic of Korea; 6 Department of Cognitive Science, Yonsei University, Seoul, Republic of Korea; PLOS: Public Library of Science, UNITED KINGDOM

## Abstract

We explore potential cross-informant discrepancies between child- and parent-report measures with an example of the Child Behavior Checklist (CBCL) and the Youth Self Report (YSR), parent- and self-report measures on children’s behavioral and emotional problems. We propose a new way of examining the parent- and child-report differences with an interaction map estimated using a Latent Space Item Response Model (LSIRM). The interaction map enables the investigation of the dependency between items, between respondents, and between items and respondents, which is not possible with the conventional approach. The LSIRM captures the differential positions of items and respondents in the latent spaces for CBCL and YSR and identifies the relationships between each respondent and item according to their dependent structures. The results suggest that the analysis of item response in the latent space using the LSIRM is beneficial in uncovering the differential structures embedded in the response data obtained from different perspectives in children and their parents. This study also argues that the differential hidden structures of children and parents’ responses should be taken together to evaluate children’s behavioral problems.

## Introduction

How children think of themselves may not be consistent with how their parents think about themselves. Reportedly, parents’ evaluations of their children are often biased, sometimes superficial, and affected by their relationship with their children. Similarly, children often do not view themselves objectively [1–5].

Parent review and child review are frequently utilized in assessing children’s behavior or psychology [6–9]. Among many scales, the Child Behavior Checklist (CBCL) [7] and the Youth Self Report (YSR) are widely used scales to evaluate children’s behavior problems, which are based on parent reports (CBCL) and self (children) reports (YSR) on the same questionnaires. To evaluate children, the CBCL and YSR comprise items of eight domains (often called syndromes): Aggressive Behavior (AB), Anxious/Depressed (AD), Attention Problems (AP), Rule-Breaking Behavior (RBB), Somatic Complaints (SC), Social Problems (SP), Thought Problems (TP), and Withdrawn/Depressed (WD). These domains are often categorized into scores of internalizing (a combination of AD, WD, and SC) and externalizing problems (a combination of RBB and AB).

The large discrepancies between the CBCL and YSR data are a commonly discussed issue in the literature [2, 6, 10–12]. To study the discrepancies between parent- and self-report measures from the CBCL and YSR, two types of approaches are conventionally taken: the direct comparison and the model-based approaches. For the direct comparison, researchers typically utilized the measures that estimate the dependency between items of two different scales, using Pearson correlation or Cohen’s Kappa [2, 10, 12, 13]. However, these direct comparison methods do not take into account the potential dependency among items that may differently exist in the two sets of measures on children. Consequently, comparison between CBCL and YSR based on the responses without considering intrinsic item inter-dependency is questionable. Three types of inter-dependent hidden structures may exist in the group item response data; between items and items, between respondents and respondents, and between items and respondents. These inter-dependent structures may differ between CBCL and YSR.

To consider item-item inter-dependency, factor analysis [7, 14, 15] has been applied to evaluate the CBCL and YSR relationship by modeling item response data with linear combinations of latent factors. Not being based on the item-response theory, those latent variables do not directly reflect the innate item or respondent characteristics embedded in the choice behaviors.

Several studies exist that defined the characteristics of items based on the item-response theory model to compare the characteristics between the two groups of respondents in the latent space [2, 12, 16–19]. Although the item-response theory model includes latent variables for items and respondents to explain group response data (see Eq 2 in the Method section), the direct item-item, respondent-respondent, or item-respondent relationships are not easily discernible. Furthermore, the previous item-response theory model may not directly evaluate each individual’s tendency toward items. Evaluating item response characteristics in each individual is critical in the practical application. Looking at individual-level discrepancies between a child-parent couple enables to generate personalized feedback on the child and parent. When exploring how a pair of child and parent views the child’s behavior differently, it would be intuitive if the items and each respondent (child and parent) have their positions (thus can be visualized) in the same latent space and if the distance between each item and respondent is explicitly defined.

In this study, to explore the multi-dimensional inter-dependent structures between items, respondents and item-respondent pairs differently embedded in the CBCL and YSR responses, we approached the problem in the latent space with a newly developed Latent Space Item Response Model (LSIRM) [20].

Latent spaces, or in other words, interaction maps, are commonly utilized to represent the relationship or dependencies between actors in various literature, such as social network analysis [21, 22], recommendation [23, 24]. The LSIRM assumes the inter-dependency can be modeled with ‘the distance’ in latent space in which both respondents and items have their ‘latent positions’ where the short distances in the latent space implies the strong dependency. For example, two items closer to each other in a latent space have stronger dependency than two items far apart. Two items with strong dependency indicate that respondents tend to show a similar response pattern to those items. Similarly, two respondents close to each other have strong dependencies, meaning that they tend to show a similar pattern of responding to test items (i.e., if one respondent responds positively to some items, the other respondent is likely to respond positively to those items). We will show that examining these dependency patterns in the two sets of measures can enlighten and shed new light on understanding the differences between CBCL and YSR. We will further present that the currently proposed method has an advantage in directly exploring respondent tendency toward items at the individual level, making it possible to examine different views in a child-parent pair on the child’s behaviors.

The rest of the paper is organized as follows: We begin by describing the empirical data example that we will use in this study, followed by conventional analyses of the data. We then briefly describe the LSIRM and show how this model can capture the dependency in item response data. Next, we describe our strategy for comparing children and parent reports from the CBCL and YSR with LSIRM. In the Result and Discussion sections, we present and discuss our analysis results, compared with conventional analysis methods, and the differential views about the children’s behaviors from the children’s and their patients’ perspectives.

## Materials and methods

### Empirical data example

#### Data

We used a dataset from the Children Mind Institute’s healthy brain network MRI database (https://childmind.org/data-sharing-initiatives/) [25]. Among 1,479 children, we selected 662 children (male: 397, female: 265) who had both the CBCL and YSR scores. Their age ranged from 10–18 years (mean: 13.8 years, standard deviation: 1.97 years). The CBCL and YSR tests consist of 120 items stating identical children’s behavioral problems in both tests. Among them, 118 items are multiple-choice questions such as “Acts too young for his/her age.” Respondents are asked to choose an option among the three response categories, “Not true,” “Somewhat or sometimes true,” “Very true or often true”, which are coded 0, 1, and 2, respectively. The other two free response items such as “Please write in any problems that were not listed above” is not considered here. The CBCL and YSR items are categorized into eight syndromes. We provided each item’s syndrome membership in the S1 Table. Several items do not belong to any particular syndromes. Those include Items 6, 7, 15, 24, 44, 49, 53, 55, 59, 60, 73, 74, 77, 80, 88, 92, 93, 98, 106, 107, 108, 109, and 110.

For data analysis, we dichotomized the original responses by combining the two positive categories of “Somewhat or sometimes true,” “Very true or often true,” and contrasting it with the only negative category of “Not true.” Such data dichotomization is not uncommon in the CBCL and YSR analysis, in part because of the frequently reported low reliability of the response categories [26–29]. After dichotomization, the mean proportion of positive responses was 0.43 with a standard deviation (SD) of 0.24 in the YSR, while in the CBCL, it was 0.27 with an SD of 0.17.

#### Analysis of CBCL and YSR differences using conventional methods

To compare with the current LSIRM analysis, we applied four analysis methods commonly used in the literature to compare the CBCL and YSR: three direct comparisons using pairwise measures, i.e., Pearson correlation, Kappa coefficients, and Jaccard similarity, and one model-based approach using item factor analysis. First, we evaluated the Pearson correlations between the CBCL and YSR at the syndrome level, using the syndrome-specific sum scores. The syndrome-level sum score is the sum of binary responses to the individual items, which can be treated as continuous data. Overall, the Pearson correlation was low, ranging from 0.23 (thought problem; TP) to 0.47 (rule-breaking problem; RBB). Table 1 shows the CBCL-YSR correlations for all syndromes. The size of the correlations was similar to the reports in the literature (e.g., [13]).

**Table 1:** Syndrome-level correlations and Kappa coefficients between CBCL and YSR.

Syndrome	Correlation	Kappa
AP	0.30	0.20
RBB	0.47	0.28
AB	0.40	0.20
WD	0.38	0.22
TP	0.23	0.14
SP	0.27	0.18
AD	0.37	0.21
SC	0.31	0.18

^a^For the Kappa coefficients, we computed the syndrome mean of the item-level coefficients.

We then computed the Kappa coefficients to evaluate the degree of agreement between CBCL and YSR at the item level, following the procedure used in [12]. The Kappa coefficients were also low, ranging from -0.02 to 0.49, with a mean of 0.18. Table 1 lists the mean Kappa coefficients for all syndromes. This result is also in line with the reports in the literature (e.g., [12]).

Next, we applied conventional dyadic similarity analysis at the item level by using the Jaccard similarity measure [30]. Jaccard similarity is a dyadic similarity measure that compares the positive response counts of item pairs. Specifically, Jaccard similarity for binary vectors *A* and *B* is defined as *J*(*A*, *B*) = |*A* ∩ *B*|/|*A* ∪ *B*|. For the CBCL and YSR data, we computed the Jaccard similarity between *P* item vectors in the CBCL and YSR, respectively, where *P* are the number of items; we then subtracted the CBCL Jaccard similarity from the YSR similarity matrix. Table 2 lists the item pairs with the top 12 most significant differences in Jaccard similarity. Interestingly, none of the identified items have syndrome membership.

**Table 2:** Twelve item-item pairs are showing the most significant differences in Jaccard similarity between the CBCL and YSR. [] indicates syndromes that the items belong to. [X] indicates that the item is not a member of any particular syndrome. For all pairs, the YSR showed high similarity (mean 0.963), while the CBCL showed low similarity (mean 0.014).

	Paired items
pair 1	107. Wets self during the day [X] ↔ 59. Plays with own sex parts in public [X]
pair 2	109. Whining [X] ↔ 59. Plays with own sex parts in public [X]
pair 3	59. Plays with own sex parts in public [X] ↔ 15. Cruel to animals [X]
pair 4	107. Wets self during the day [X] ↔ 92. Talks or walks in sleep [X]
pair 5	92. Talks or walks in sleep [X] ↔ 59. Plays with own sex parts in public [X]
pair 6	109. Whining [X] ↔ 107. Wets self during the day [X]
pair 7	59. Plays with own sex parts in public [X] ↔ 6. Bowel movements outside toilet [X]
pair 8	98. Thumb-sucking [X] ↔ 59. Plays with own sex parts in public [X]
pair 9	107. Wets self during the day [X] ↔ 6. Bowel movements outside toilet [X]
pair 10	107. Wets self during the day [X] ↔ 98. Thumb-sucking [X]
pair 11	109. Whining [X] ↔ 98. Thumb-sucking [X]
pair 12	88. Sulks a lot [X] ↔ 59. Plays with own sex parts in public [X]

Lastly, we applied exploratory item factor analysis to the CBCL and YSR data, using the R package ‘mirt’ [31]. In the item factor model, the probability of answering positively to item *i* for respondent *k* is defined as follows:

$$\begin{array}{c} \text{logit}\left(P\left(y_{ki}=1\right)|\alpha_{i},\theta_{k},d_{i}\right)={\alpha_{i}}^{T}\theta_{k}+d_{i} \end{array}$$ (1)

where *d*_*i*_ is item intercept, *α*_*i*_ is item-specific latent factor, and *θ*_*k*_ is respondent-specific latent factor [31].

In this experiment, we found the optimal number of factors was four, both for the CBCL and YSR data, based on G2 goodness-of-fit statistics and their p-values [32]. The factor structure comparison table and their fit statistics are given in the Supplementary S2 Table. The factor structure, however, was different between the two datasets. To summarize, for the first two factors, the CBCL and YSR show similar loading structures: the first significant factor covers most items of the AD syndrome (internalizing syndrome), and the second important factor covers most items of the RBB syndrome, an externalizing syndrome. However, the last two factor structures are quite different between the CBCL and YSR. The third factor is loaded on attention, social and thought problems (AP, SP, and TP) syndromes in the CBCL, while in the YSR, it is loaded on SC and WD syndromes. The fourth factor is loaded on the items with no syndrome membership in the YSR, while in the CBCL, this factor is loaded on most of the AB syndrome. These results are also consistent with the literature [6, 14].

All four analyses point to that there are significant discrepancies between the CBCL and YSR data. We will later compare and discuss the proposed latent space approach’s results compared with these conventional methods. These conventional methods work as a reference for validating the proposed algorithm and providing a rationale for applying the current model-based approach.

### Latent space item response model

The LSIRM [20] has been developed as an extension of the Rasch model to alleviate the conventional assumption of conditional independence (item responses are independent given a latent trait) and of homogeneity (respondents with the same trait level have equal success probability to an item). The LSIRM introduces latent positions, ***w*_*i*_** and ***z*_*k*_** for each item *i* and each respondent *k* in a *d*-dimensional latent space. The probability of positive response $P(y_{ki}=1)$ to an item *i* from a respondent *k* is then formulated as follows:

$$\begin{array}{c} \text{logit}\left(P\left(y_{ki}=1\right)|\beta_{i},\theta_{k},w_{i},z_{k}\right)=\beta_{i}+\theta_{k}-\left|\right|w_{i}-z_{k}\left|\right|, \end{array}$$ (2)

with item coefficients *β*_*i*_ (item easiness), respondent coefficients *θ*_*k*_ (latent trait), and the latent positions of respondent ***z*_*k*_** and item ***w*_*i*_** ($z_{k},w_{i}\in R^{d}$). The latent positions of respondent ***z*_*k*_** and item ***w*_*i*_** are determined by their distances, given the respondent and item coefficients. The resulting latent space approach provides an interaction map that represents the interactions of respondents and items, and helps derive insightful diagnostic information on items as well as respondents. We will use the words latent space and interaction map simultaneously. Though the dimension of the latent space or the distance measure can be arbitrarily chosen by researcher, we stick to $R^{2}$ and euclidean distance as Jeon et al. [20] remarked because of its strength in visualization. Eq 2 explains that when a respondent is further away from an item (i.e., larger distance and weaker dependency), the probability of giving a positive response to the item decreases. When a respondent becomes closer to an item (i.e., the shorter distance, the stronger dependency), the probability of giving a positive response increases. Note that Eq 2 is slightly different from Jeon et al. [20]’s formulation in that we fix the distance weight as one. This set-up enables us to match the two interaction maps from the CBCL and YSR data.

#### Transitivity

**Fig 1:**
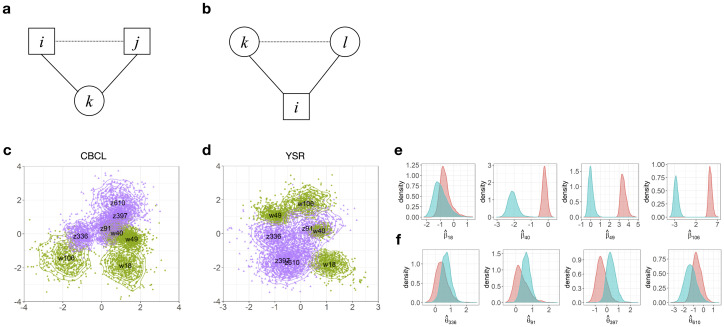
The concept of the distance, position and the latent space (interaction map). (a) and (b) visualize the triangle inequality of the distance. (a) *k* is a respondent, and *i* and *j* are two items. If the positions of two items *i* and *j* are close to respondent *k* in the interaction map, the two items are fairly close to each other, by the triangle inequality. (b) *i* is an item, and *k* and *l* are two respondents. If two respondents *k* and *l* are close to the item *i*, the two respondents *k* and *l* are also close. (c) is an example of the posterior distributions of latent positions in the CBCL interaction map, and (d) is the posterior distributions in the YSR interaction map. For visualization, only four items (w18, w40, w49, w106) and four respondents (z91, z336, z397, z610) are displayed. Green dots and lines around the item positions indicate the posterior distributions of the item positions. Purple dots and lines around the person positions indicate the posterior distributions of the person positions. Note that the interaction maps differ for parents (CBCL) and children (YSR). Note also that both item and person positions are located in the same map. The short distance between two latent positions of items (respondents) in the interaction map implies a large dependency between two items (respondents). (e) and (f) present posterior distributions of selected item coefficients (e) and respondent coefficients (f). Blue indicates the CBCL, and red indicates the YSR distributions for each coefficient. All Bayesian inferences are made through these posterior samples; thereby flexible inference such as variance or overlapped portion with other distribution can be made.

Note that although the model specifies distances between respondents and items in Eq 2, the model also captures item-item and respondent-respondent distances. Fig 1(a) and 1(b) explains how it is possible; (a) if a *k*-th respondent responds positively to an *i*-th item and at the same time responds positive to a *j*-th item, the latent positions of ***w*_*i*_** and ***w*_*j*_** are likely to be close to each other; (b) if an *i*-th item is positively answered by respondents *k* and *l*, ***z*_*k*_** and ***z*_*l*_** are likely to be close to each other in interaction map. This is a property interaction map referred to as transitivity [33, 34].

#### Dependency

The distance between two latent positions in latent space is the measure of dependency. The short distance between *i*th item latent position (*w*_*i*_) and *j*th item latent position (*w*_*j*_) means high dependency between two items. The respondent who responds positively to item *i* is likely to respond positively to *j*th item, vice-versa. The LSIRM models the item-item, respondent-respondent, and item-respondent dependency by projecting respondents and items to the continuous latent space. Therefore, the model-based comparison of two scales from different informants using the LSIRM allows us to evaluate pairwise dependencies between items, between respondents, and between items and respondents. Additionally, comparisons in aspects of higher-order dependencies, such as clustering patterns and relationships between clusters, are available using the LSIRM.

Fig 1(c) and 1(d) illustrate the interaction maps of the CBCL and YSR for the selected respondents and items for specific example. The figures display the posterior samples of latent positions for the selected items and respondents. For the item-item comparison, Items 40 and 49 (w40, w49) are relatively close in both interaction maps. This implies that Items 40 and 49 have a high dependency in both YSR and CBCL responses. On the other hand, Items 49 and 106 (w49, w106) are apart more in the CBCL interaction map than in the YSR interaction map, which implies a difference in dependency between the YSR and CBCL responses. Note that Item 40 states “Hears sounds or voices that aren’t there”, Item 49 states “Constipated, doesn’t move bowels”, and Item 106 is about “Vandalism”. Therefore, we can identify the differences of inherent dependency between pairs of items by comparing the interaction maps of the CBCL and YSR. For example, parents tended to make different reports on vandalism and constipation, while children responded similarly to those topics.

Regarding the respondent-respondent relationship, Fig 1(c) and 1(d) also show that some respondents are close to their locations, while others are further away. For example, Respondents 610 and 397 (z610, z397) are close to each other in both interaction maps, meaning that their response patterns were similar, given their latent trait levels and item difficulty. Similarly, for the respondent-item, we can also observe that Respondent 236 (z236) is closer to Item 106 (w106) in the CBCL space than in the YSR space. This indicates that for Respondent 236, the child’s probability of positively responding to “Vandalism” was lower than her parent’s likelihood of giving positive responses.

Of note, the current model assumes some hidden tendencies in the parents’ responses toward their children. Thus, other parent respondents’ responses are needed to decompose those hidden parent-to-children tendencies as a structure, even though the parent respondents are not directly related to the other children.

#### Estimation

To estimate the LSIRM parameters, we used a Bayesian approach, following [20]. The priors of *β*_*i*_, *θ*_*k*_, ***z*_*k*_** and ***w*_*i*_** are set to an independent normal distribution with a mean of 0 and some variances. The hyper-prior for the variance parameter of *θ*_*k*_ (*k* = 1, ⋯, *N*) is set to be a conjugate inverse Gamma distribution:

$$\begin{array}{cc} \beta_{i}|\tau_{\beta }^{2} & ∼N(0,\tau_{\beta }^{2}),\\ \theta_{k}|\sigma^{2} & ∼N(0,\sigma^{2}),\\ \sigma^{2} & ∼\text{Inv-Gamma}\left(a_{\sigma },b_{\sigma }\right),a_{\sigma }>0,b_{\sigma }>0,\\ w_{i} & ∼{\text{MVN}}_{d}\left(0,I_{d}\right),\\ z_{k} & ∼{\text{MVN}}_{d}\left(0,I_{d}\right). \end{array}$$

Metropolis-Hasting-within-Gibbs sampler [35] was used, which generates the posterior samples of *β*_*i*_, *θ*_*k*_, ***z*_*k*_**, and ***w*_*i*_** for *k* = 1, ‥, *N* and *i* = 1, ‥, *P*. Additional details of the estimation procedure are provided in the S1 Appendix. We set the number of iterations to 30,000 and take every 5-th sample after the first 5,000 steps as a burn-in period. Convergence was satisfactory, and the posterior distributions of the example item and person parameters are presented in Fig 1(e) and 1(f).

Due to the invariance property of the distances (invariance to rotation, reflection, and translation), multiple solutions may be available for the latent positions that produce the same distance matrix. This issue is common for models that involve latent spaces [21]. To resolve such an identifiability issue, we applied Procrustes transformation [36] as a post-processing procedure, which is a standard method to resolve latent position identifiability in the literature [21, 22]. For [*Z*] the class of positions equivalent to *Z* under rotation, reflection, translation, the Procrustean transformation is Z* = argmin_*TZ*_tr(*Z*_0_ − *TZ*)^*T*^ (*Z*_0_ − *TZ*), where *Z*_0_ is a fixed set of positions and *T* ranges over the set of rotations, reflections, and translations. It is known that Z* is the closest element to *Z*_0_ in terms of the sum of squared and is unique if *Z*_0_*Z*^*T*^ is nonsingular [37]. Here the target *Z*_0_ would be the positions draw that produces maximum a posteriori and the other posterior draw of latent positions are carried out Procrustes transformation to resolve the invariance to reflections, rotations and translations. All Bayesian inferences are made through the posterior samples obtained after the post-processing.

To deal with missingness in the datasets under investigation, we extended the estimation procedure of Jeon et al. [20] with Bayesian data augmentation [38]. Specifically, we imputed the missing data with the posterior samples, where an estimated item response for missing ${\hat{y}}_{ik}$ was generated using the estimated parameters of the previous step of the Gibbs sampling. The imputed response values were updated in every Gibbs sampling step. After assuming missing at random (MAR), this Bayesian data augmentation produces valid imputation results [39]. Of all item and respondent pairs, the missing proportion was 0.192% in YSR and 0.259% in the CBCL. We made MAR assumption because currently there is no established method for testing the nature of missingness, and MAR is often assumed in the CBCL and YSR analysis. Further, addressing potential non-ignorable missingness is not the primary purpose of the current study. We will look into the sensitivity of this assumption in future research.

### Strategies for analyzing the CBCL and YSR differences with the LSIRM

We applied a series of further analyses for model-based examinations of the CBCL-YSR differences with the discussed latent space approach.

First, we fit the separate LSIRM to the CBCL and YSR datasets and estimated two sets of item coefficients *β*_*i*_, respondent coefficients *θ*_*k*_, and their latent space positions (***z***_***k***_ and ***w***_***i***_). We evaluated the goodness of fit of the model to each dataset.

Second, we examined the differences between the item coefficients *β*_*i*_ estimated from the CBCL and YSR analysis. We also compared item positions ***w*_*i*_** in the CBCL and YSR interaction maps and checked whether the items with a large discrepancy between CBCL and YSR in item coefficients *β*_*i*_ show distinct interaction patterns between them.

Third, we compared the CBCL and YSR in terms of item-pair distance or item-item dependency over the interaction map. For this, we first evaluated the distance distribution of the two items *i* and *j* (i.e., the posterior distributions of the distance ||***w*_*i*_** − ***w*_*j*_**||) in the CBCL denoted as $D_{ij}^{CBCL}\left(x\right)$ and YSR denoted as $D_{ij}^{YSR}\left(x\right)$, respectively. This posterior distribution function $D_{ij}^{CBCL}$ and $D_{ij}^{YSR}$ were obtained by calculating the Euclidean distance of each posterior sample of *w*_*i*_ and *w*_*j*_ in CBCL and YSR, obtained by fitting the LSIRM. This distribution represents the estimated dependency between item *i* and item *j* in terms of the distribution. We then assessed the significance of the differences of each item-pair dependency by evaluating the overlapped portion of their distance distribution. The overlapped portion was defined as

$$\begin{array}{c} R=\int_{-\infty }^{\infty }\text{min}\left(D_{ij}^{CBCL}\left(x\right),D_{ij}^{YSR}\left(x\right)\right)dx, \end{array}$$

where $\text{min}(D_{ij}^{CBCL}\left(x\right),D_{ij}^{YSR}\left(x\right))$ indicates the overlapping areas of the two distributions. *R* ≤ .05 indicates that the two distributions are fairly different in a statistical sense [40–42]. An alternative approach, the calculation of the Kullback-Leibler (KL) divergence between $D_{ij}^{CBCL}\left(x\right)$ and $D_{ij}^{YSR}\left(x\right)$, was also applied to capture the difference of item-pair dependency and its results were reported in the Supplementary S2 Appendix.

Fourth, item syndrome-level dependency is evaluated, with the syndrome positions identified with the centroid of the syndrome-specific items. Since the item syndrome is named with its semantic properties, it makes the axis of the interaction map more interpretable and comparable with intuition. To highlight the meaning of this axis, we used cosine similarity to measure the similarity of item syndrome level. The cosine similarity is measured based on the coordinates of the syndrome latent positions in the CBCL and YSR separately. The cosine similarity of two vectors ***a*** and ***b*** can be computed as

$$\begin{array}{c} \begin{array}{c} \text{cos}(\phi )=\frac{a^{⊤}\;b}{{\left||a|\right|}_{2}{\;||b||}_{2}}, \end{array} \end{array}$$

where *ϕ* is the angle between two vectors ***a*** and ***b***, and ||***a***||_2_ > 0 and ||***b***||_2_ > 0 are their lengths. cos(*ϕ*) ranges from -1 to 1, while -1 indicates that the two vectors point to the opposite directions, and 1 indicates the same direction. Cosine similarity of 0 means that the two vectors are orthogonal.

Fifth, we evaluated differences in the dependency between respondents with their syndrome levels. We presented that the distance between each respondent and syndrome in the interaction map could be used to find the vulnerable syndromes for each respondent. We then compared K-means clustering of all respondents based on the distance from all item syndromes and demonstrated how the interaction map approach could derive unique findings.

## Results

To evaluate the goodness of fit of the LSIRM to CBCL and YSR data, we assessed the proximity of the predicted item responses based on the estimated model to the original item responses, which is a commonly used strategy for evaluating the prediction accuracy of binary classification [43–45]. As evaluation criteria, we used sensitivity, specificity, and overall accuracy, which are reported as reliable measures [46, 47].

The three indices are defined as $\text{Specifity}=\frac{\text{TN}}{\text{TN}+\text{FP}}$, $\text{Sensitivity}=\frac{\text{TP}}{\text{TP}+\text{FN}}$, Overall $\text{accuracy}=\frac{\text{TP}+\text{TN}}{\text{TP}+\text{TN}+\text{FP}+\text{FN}}$, where TN is true negative, TP is true positive, FP is false positive, and FN is false negative. Table 3 shows the result. All values are higher than 0.70, except for the sensitivity for the CBCL. This suggests that overall, the LSIRM showed satisfactory fit to both the CBCL and YSR data in terms of prediction.

**Table 3:** Three goodness-of-fit measures of the LSIRM to the CBCL and YSR data.

	CBCL	YSR
Specificity	0.927	0.846
Sensitivity	0.556	0.720
Overall accuracy	0.881	0.876

### Item coefficients and positions

**Fig 2:**
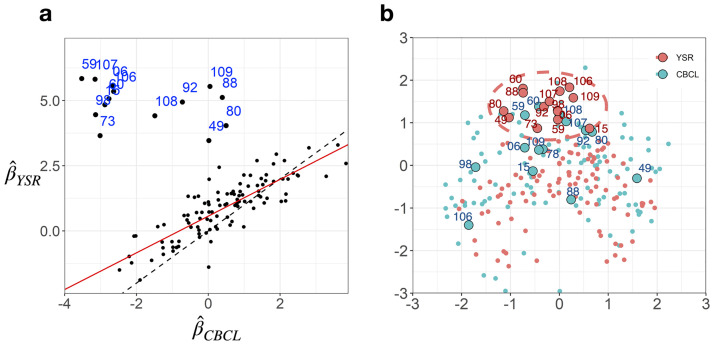
Item-wise comparison between the CBCL and YSR. (a) Comparison between the CBCL and YSR item coefficients ${\hat{\beta }}_{i}$ is displayed. The red line is the linear regression line between the CBCL and YSR estimates. The dotted line is *y* = *x*. Numbers indicate the outliers that deviate largely from the linear trend. For most test items, the two sets of ${\hat{\beta }}_{i}$ are similar with the correlation of *r* = 0.83. However, the indicated items do not follow this general pattern. (b) An integrated interaction map for the CBCL and YSR item positions is presented. Blue and red dots represent the item positions identified in the CBCL and YSR data, respectively. Larger dots with number labels are the outlier items identified in (a). Note that the distance between dots indicates the degree of association of the two items in the latent space. For the close items, if respondents respond positively to an item, they are more likely to respond positively to the other item.

Fig 2(a) shows the relationship between the CBCL and YSR in the estimated item coefficients ${\hat{\beta }}_{i}$. For most test items, the two sets of ${\hat{\beta }}_{i}$ are similar with the correlation of r = 0.83. However, a group of items does not follow this general pattern (marked in blue in the scatter plot). For those items, ${\hat{\beta }}_{YSR}$ were higher than ${\hat{\beta }}_{CBCL}$, meaning that the children than their parents more easily endorsed them. The specific contents of those items were listed in Table 4. Those items appear to address behaviors related to sexual or physiological problems. That is, parents tended to believe that their children did not have sexual and physiological problems, even when the children themselves acknowledged such problems. Further analysis of these items with the interaction map can lead to more detailed interpretation.

**Table 4:** Items that differ between the CBCL and YSR in item-wise coefficients ${\hat{\beta }}_{i}$ or item easiness (a tendency of the positive answer). The item easinesses of the YSR for these items are greater than the CBCL (${\hat{\beta }}_{CBCL}<{\hat{\beta }}_{YSR}$).

Questions	
6. Bowel movements outside toilet	88. Sulks a lot
15. Cruel to animals	92. Talks or walks in sleep
49. Constipated, doesn’t move bowels	98. Thumb-sucking
59. Plays with own sex parts in public	106. Vandalism
60. Plays with own sex parts too much	107. Wets self during the day
73. Sexual problems	108. Wets the bed
80. Stares blankly	109. Whining

Fig 2(b) shows interaction maps with item latent positions ***w*_*i*_** obtained from CBCL and YSR analysis with respective LSIRMs. For visual comparisons, the two estimated interaction maps were matched and integrated using Procrustes matching. Blue dots indicate the positions of test items from the CBCL space, and red dots indicate the positions of test items in the YSR space. Respondents’ positions are not presented in Fig 2(b) to focus on item position comparisons.

The distributions of item positions were generally similar between CBCL and YSR spaces, but there were some notable exceptions. The aforementioned items with dissimilar item coefficients (items in Fig 2(a) and Table 4) were placed in different regions of the CBCL and YSR interaction maps. See items marked with numbers in Fig 2(b). These items were closely located to each other in YSR (marked with a dotted red oval), which was not the case in the CBCL data.

### Differences in item-item dependency

We evaluated item-pair distance differences between the CBCL and YSR data based on $D_{ij}^{CBCL}$ and $D_{ij}^{YSR}$ values. For most item pairs, the difference was statistically significant (*p* < 0.01) based on the Kolmogorov-Smirnov test. Table 5 lists the top 12 item pairs with the largest CBCL-YSR differences.

**Table 5:** Top 12 item pairs that show the largest differences between the CBCL and YSR in terms of $D_{ij}^{CBCL}$ and $D_{ij}^{YSR}$. AP, SP and TP etc. within the brackets indicate the syndrome that each item belongs.

Pairs	Items	Distance (MAP)
Pair 1	1. Acts too young for his/her age [AP]	YSR (0.472) < CBCL (2.113)
	↔ 41. Impulsive or acts without thinking [AP]	
Pair 2	100. Trouble sleeping [TP]	YSR (0.342) < CBCL (1.938)
	↔ 27. Easily jealous [SP]	
Pair 3	100. Trouble sleeping [TP]	YSR (0.745) < CBCL (3.109)
	↔ 10. Can’t sit still, restless or hyperactive [AP]	
Pair 4	100. Trouble sleeping [TP]	YSR (1.282) < CBCL (3.17)
	↔ 41. Impulsive or acts without thinking [AP]	
Pair 5	100. Trouble sleeping [TP]	YSR (0.752) < CBCL (2.964)
	↔ 78. Inattentive or easily distracted [AP]	
Pair 6	100. Trouble sleeping [TP]	YSR (0.627) < CBCL (2.535)
	↔ 79. Speech problem [SP]	
Pair 7	11. Clings to adults or too dependent [SP]	YSR (0.327) < CBCL (2.788)
	↔ 10. Can’t sit still, restless or hyperactive [AP]	
Pair 8	11. Clings to adults or too dependent [SP]	YSR (0.841) < CBCL (3.009)
	↔ 41. Impulsive or acts without thinking [AP]	
Pair 9	11. Clings to adults or too dependent [SP]	YSR (0.338) < CBCL (2.462)
	↔ 78. Inattentive or easily distracted [AP]	
Pair 10	36. Gets hurt a lot, accident-prone [SP]	YSR (2.641) > CBCL (0.346)
	↔ 12. Complains of loneliness [SP]	
Pair 11	12. Complains of loneliness [SP]	YSR (2.451) > CBCL (0.534)
	↔ 83. Stores up too many things he/she doesn’t need [TP]	
Pair 12	35. Feels worthless or inferior [AD]	YSR (2.588) > CBCL (0.809)
	↔ 47. Nightmares [SC]	

**Fig 3:**
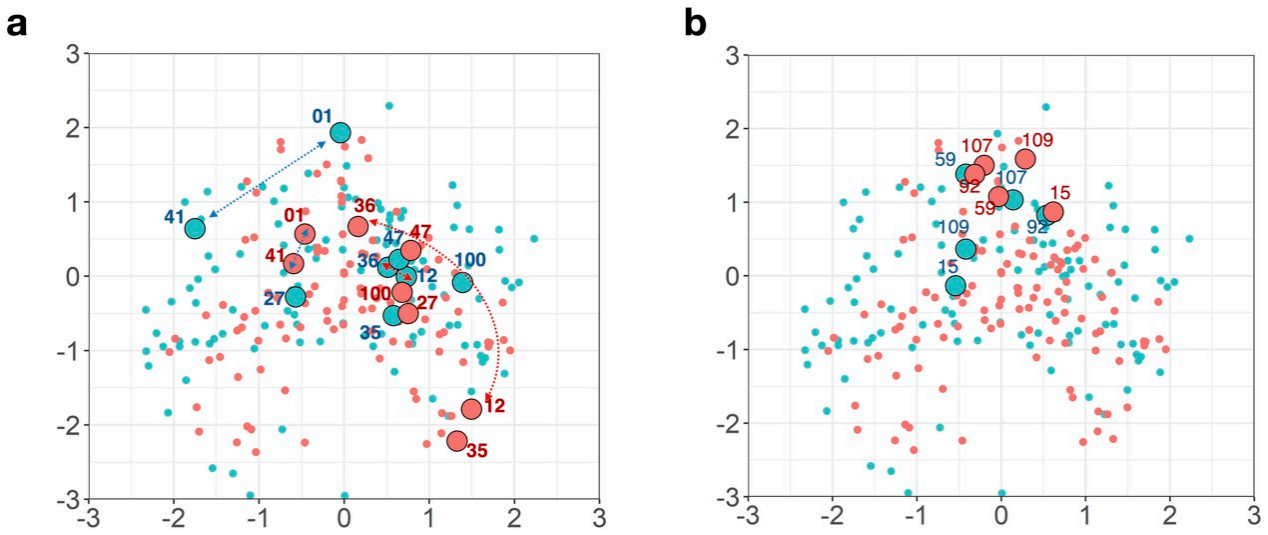
(a) Four item-item pairs with the largest differences in the distance (see Table 5). Double-side arrows are drawn to show the distances between two pairs (items 40 and 01; items 36 and 12) in the CBCL (blue) and YSR data (red). In addition to the position of each item, the distance between the two items’ positions should be noted. (b) Four item-item pairs with the largest difference in the Jaccard similarity between the CBCL and YSR data. This implies that our analysis of item-pair dependency does not offer the same kind of information as the analysis of the item coefficient and the conventional similarity analysis.

Fig 3(a) illustrates how different the item-pair distances were between the CBCL and YSR data for the top four pairs. Interestingly, these items did not match the items identified from the item coefficient (*β*_*i*_) difference analysis (Table 4). In addition, they did not match the items identified with the Jaccard similarity analysis (Table 2 and Fig 3(b)). Fig 3(b) shows the Jaccard similarity between the CBCL and YSR data. The items identified in conventional direct comparison using Jaccard similarity were not coherently located with Fig 3(a) in the interaction map. This implies that our analysis of item-pair dependency does not offer the same kind of information as the analysis of the item coefficients and the conventional similarity analysis.

With item-pair differences, two groups of pairs can be distinguished in Table 5. In one group, those pairs were believed to have similar behaviors from the children’s perspective, but not so much to the parents’ view (Pairs 1–9). In the other group, the item pairs showed the opposite patterns: while they were perceived similar behaviors to the parents’ eyes, they were believed to have different problems to the children (Pairs 10–12).

For example, to the children, “Act too young” and “Impulsive” (Pair 1) were similar behaviors, while it was not the case to the parents. The parents consider their children’s behaviors of “Complains of loneliness” and “Stores up too many things he/she doesn’t need” (Pair 11) are highly relevant, but children consider them irrelevant. Most of these items are the members of attention, social and thought problems (AP, SP, and TP) syndromes, indicating that those syndromes’ behaviors were likely to be evaluated and reported differently by different informants, i.e., parents and children in the current context.

**Fig 4:**
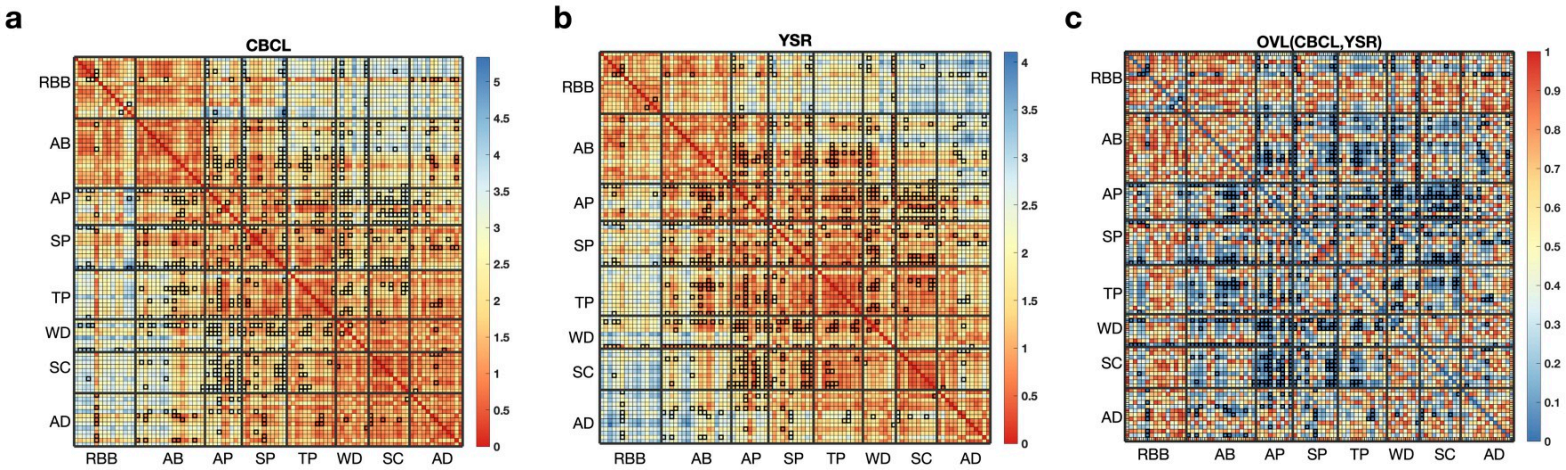
Item-item distance heatmaps of the CBCL and YSR, and an overlapped portion heatmap between the CBCL and YSR. (a) displays the distance heatmap between pairs of item latent positions in the CBCL ($D_{ij}^{CBCL}$) and (b) displays that in the YSR ($D_{ij}^{YSR}$). (c) shows the heatmap of the overlapped portion between $D_{ij}^{CBCL}$ and $D_{ij}^{YSR}$ distributions for all item pairs. The item pair with a large overlapped portion means the perceived relation of that item pair is similar in both informants, while the item pair with a small overlapped portion means their relationship is perceived as different. The item pair with a large overlapped portion is colored with red, and the item pair with a small overlapped portion is colored with blue. The item pairs with overlapped portions less than 0.05 are marked with bold edges. About 9% of the pairs had overlapping portions of less than 0.05, and most of these pairs exist between different item syndromes (off-diagonal blocks), related to attention (AP), social (SP), and thought (TP) problems.

We assessed the overall differences in item-item dependency between the CBCL and YSR data by evaluating the overlap of the $D_{ij}^{CBCL}$ and $D_{ij}^{YSR}$ distributions. Fig 4 summarizes above Fig 3 with respect to item-item dependency categorized by syndromes. The distance of each item pair (i.e., expectation of the probability distribution *D*_*ij*_) is displayed in Fig 4(a) and 4(b) for the CBCL and YSR, respectively. The low value of distance means that their latent position was located closely and had a strong interaction. Fig 4(c) summarizes the overlapped portion for all pairs of items between CBCL and YSR. The item pairs with overlapped portion less than 0.05 are marked with bold edges. Of all the item pairs, about 9% of the pairs had overlapped portions of less than 0.05. The alternative approach using KL-divergence (S2 Appendix) suggests that pairs of items with a large discrepancy in KL-divergence between CBCL and YSR are positioned differently in their interaction maps.

#### Syndrome-level distances

**Fig 5:**
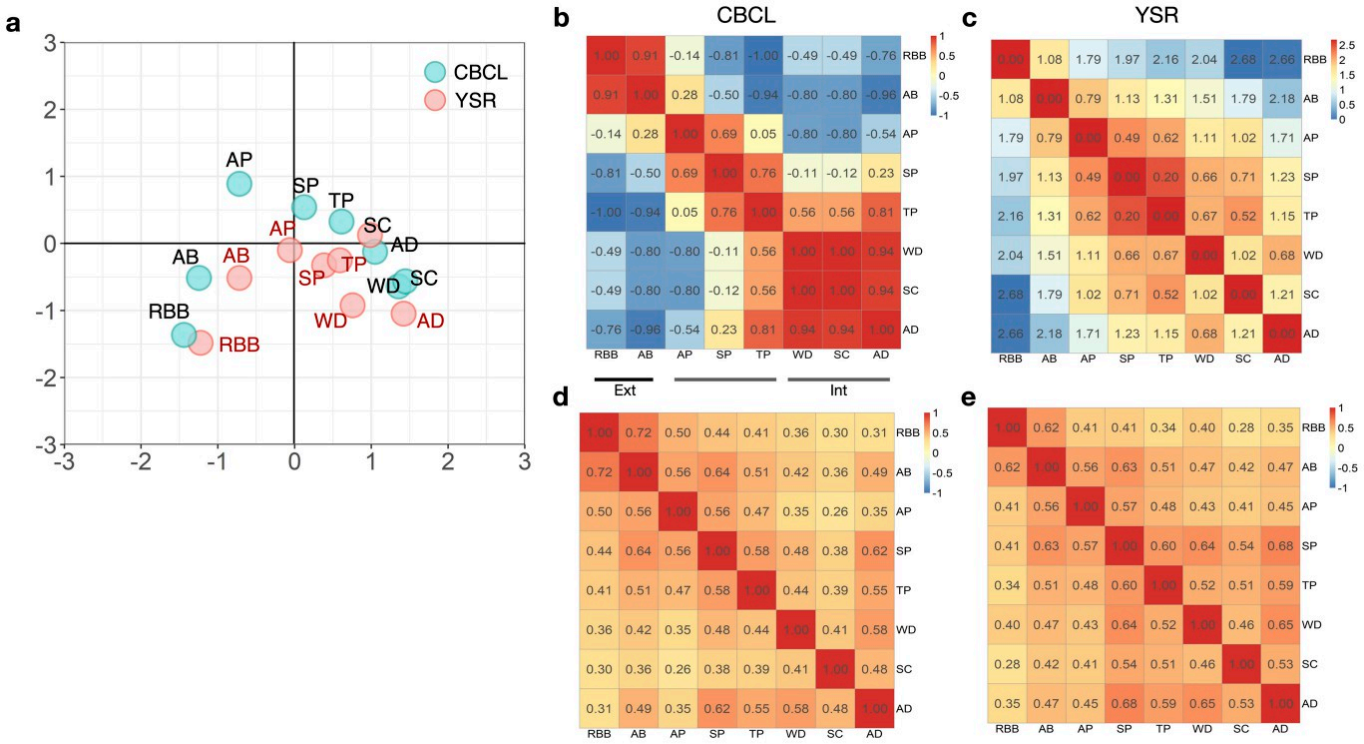
(a) Interaction map of the syndrome positions identified with CBCL (blue) and YSR (red). Recall that syndromes are denoted with their abbreviation, Aggressive Behavior (AB), Anxious/Depressed (AD), Attention Problems (AP), Rule-Breaking Behavior (RBB), Somatic Complaints (SC), Social Problems (SP), Thought Problems (TP), and Withdrawn/Depressed (WD). These domains are often further categorized into internalizing (INT, a combination of AD, WD, and SC) and externalizing problems (EXT, a combination of RBB and AB). (b) and (c) are the heatmaps of the cosine similarity matrices of the item syndrome positions in the CBCL and YSR. (d) and (e) show the heatmaps of the correlation matrices of the raw syndrome scores in the CBCL and YSR. The identified dependency is roughly similar. However, the inter-correlations between externalizing syndromes (RBB, AB) and between internalizing syndromes (AD, WD, SC) were not outstanding in (d) and (e) compared to (b) and (c).

Fig 5(a) shows the syndromes’ latent positions (identified as the centroid of the syndrome item members) from the CBCL and YSR spaces. In both spaces, externalizing syndromes (RBB, AB) are close to each other, and internalizing syndromes (AB, WD, and SC) are also close to each other, while externalizing and internalizing syndromes are far apart from each other. Fig 5(a) also shows that syndromes tend to be closer in their positions in the YSR space than in the CBCL space, meaning that the syndromes were seen more similar each other to the children’s perspective than to the parents’ view.

We measured cosine similarity between syndrome positions identified in the CBCL and YSR data. The cosine similarity is measured based on the coordinates of the syndrome latent positions in CBCL and YSR separately. Fig 5(b) and 5(c) display the heatmaps of cosine similarity measures for CBCL and YSR, and they suggest that the attention, social and thought problems (AP, SP, and TP) syndromes show different similarity patterns between CBCL and YSR. In particular, the characteristics of AP are distinct from other syndromes in the CBCL space. This result is consistent with the findings based on item-item dependency patterns.

In contrast, a simple inter-syndrome correlation analysis based on syndrome-level raw scores presented in Fig 5(d) and 5(e) did not identify inter-and intra-correlations between the syndromes. For example, the inter-correlations between externalizing syndromes (RBB, AB) and between internalizing syndromes (AD, WD, SC) were not outstanding.

### Respondent distances to syndromes

**Fig 6:**
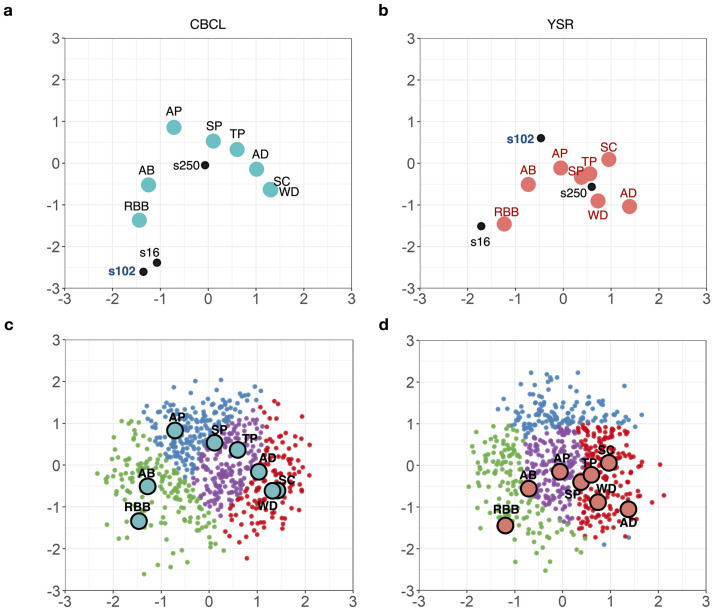
(a) and (b) display the syndrome positions and three respondents (s16, s102, s250) in the CBCL and YSR space, i.e., couples of parents (CBCL) and children (YSR), respectively. Note that both latent variables for items and latent variables for respondents are plotted in a latent coordinate. Latent positions for syndromes are presented as colored circles with solid black borders, and respondents’ latent positions are displayed as dots. Three respondents are exemplary selected based on their latent positions in the CBCL and YSR: the two respondents located in the center (s250) and in the boundary (s16) show similarity between children and parents but s102 show difference in the latent positions between children and parents. (c) and (d) show the K-means clustering results of all respondents based on the distance from all item syndromes in the CBCL and YSR spaces.

Individual respondents’ distances to the behaviors or the syndromes in the interaction map are useful as they are the basis to draw diagnostic conclusions about the respondents. To illustrate differences and similarities in the respondents’ distances to the syndromes, we selected three respondents as an example (s16, s102, and s250) in Fig 6(a) and 6(b). Respondent s16 was closest to the syndromes RBB and AB in both the CBCL and YSR spaces, indicating that she/he were more likely to show the behaviors of the RBB and AB syndrome than other behaviors, and such a diagnosis of s16 would be similar based on the CBCL, and YSR reports. On the other hand, respondent s102 was located somewhat differently in the two spaces. This student was close to RBB and apart from AP in the CBCL space, but she/he was closer to AP than RBB in the YSR space.

Finding vulnerable syndromes using an interaction map is different from the conventional approach, such as simply comparing the positive response counts of syndromes for each respondent, because the latent position in the LSIRM is assigned by considering the dependency between all other objects. For example, one could draw the same conclusion with Fig 6(b) using simply comparing the positive response counts for s16, the respondent who answered about 2.5 times more positively to externalizing syndromes (23 positive responses) than internalizing syndromes (9 positive responses) in the YSR. On the other hand, s16 in the CBCL answered in externalizing and internalizing syndromes quite similarly (20 and 18 positive responses, respectively.) but is still located close to the RBB and AB, the externalizing syndromes in the interaction map, Fig 6(a). This is because the other respondents with similar response patterns to s16 in terms of each item, not discrete syndrome-level, responded more positively to externalizing syndromes. Therefore, although s16 responds similarly to both internalizing and externalizing syndromes in the CBCL, s16 might be more vulnerable to externalizing syndromes when considering other respondents with similar patterns. This means that one could end up with different conclusions about their most likely behaviors and the existence of cross-informants discrepancy depending on whether the dependency was considered or not.

We applied K-means clustering to the respondent positions to see the differences in the respondent-syndrome distances between the CBCL and YSR spaces. The results are shown in Fig 6(c) and 6(d). Four clusters were identified, each with the CBCL and YSR respondent positions (distinguished with four colors). However, the four clusters identified from the two spaces do not necessarily match in terms of their most likely syndromes, given clusters’ different distances to the syndromes. For example, the cluster located in the upper side of the interaction map (blue) did not appear close to any particular syndrome in the YSR space (Fig 6(d)), while such cluster did not exist in the CBCL space (Fig 6(c)). This re-confirms that using either parent’s or children’s reports can lead us to a different conclusion about the children’s most likely syndromes and behavioral problems.

## Discussion

This paper discusses a new way of exploring the hidden structure of item responses from different informants with a latent space modeling approach, referred to as the LSIRM method. This new methodology was applied to the comparative analysis of the CBCL and YSR, which are widely-used multi-item scales on children’s behavioral and emotional problems, based on parent- and self-reports, respectively. The LSIRM analysis spotted substantial differences between the CBCL and YSR measures which were not examined with existing methods, either direct comparison approaches or model-based item factor analysis.

Direct comparison analysis using Pearson correlation, Cohen’s Kappa, or Jaccard similarity, computes the similarity between each item (or between each syndrome, with the sum of items belonging to it) pair-wisely and thereby treat each subject independently. This type of direct item-wise approach does not consider the potential dependency that may exist in other items or other respondents and cannot be used to compare the dependency between items and respondents at the same time. To figure out the item-respondent relationship, additional analysis such as linear regression or subject grouping is needed [2, 12, 18, 19]. We do not disregard the possibility that some of those dependencies can be explored by extending the conventional methods. For example, the residual covariance matrix may be used to find item-item dependencies. We have found that conventionally used direct comparison methods partly capture the relationship inherent in data more obtusely but sometimes lead to different conclusions (e.g., Fig 3). The consistency between the results of the LSIRM and direct comparison methods reported in the previous literature and this study supports the validity of the proposed algorithm. This validity is critical for the LSIRM, which has inherent overfitting problems as a complex model. It also lays the basis for the new findings’ reliability derived from the further analysis unique to the LSIRM.

Item factor analysis [48, 49], shown in Eq 1, is designed to identify the latent factor structure underlying the item response data by assuming latent factors to be linear combinations of observed item responses. The item clusters identified with item factor analysis (S2 Table) are roughly similar to the LSIRM, showing high consistency between the CBCL and YSR in the internalizing and externalizing syndromes. That is, item factor analysis can identify clustering of items similar to the LSIRM. More specifically, the item factor analysis can capture item-by-respondent interactions to some degree in the sense that factor loadings (specific to items) are multiplied by factors (specific to respondents) (See Eq 1). Nevertheless, not all item-by-respondent interactions can be quantified with the item factor analysis because factor loadings are item-specific, not item-by-respondent specific. No parameters represent interactions between items and respondents in the item factor analysis model (Eq 1). Furthermore, the item factor analysis cannot capture the similarity of respondents in terms of response patterns. In contrast, the LSIRM explicitly models item-by-respondent interactions in the form of distances in a metric space, which makes it possible to examine similarities among items and similarities among respondents (Eq 2). As exampled in Fig 6, the characteristics of each individual can intuitively be represented in terms of the respondent’s position and distances from items in the LSIRM. This model enables the investigation of each child and parent pair precisely. Of note, in terms of model parameter estimation, we used a Bayesian estimation with uninformative priors; thus, it is less likely that the model inversion scheme based on Bayesian or frequentist approaches may not be a significant factor in deriving the results.

The LSIRM differentiates between each item’s coefficient and item position in the latent space. The item coefficient is estimated independent of other items and individual characteristics. In contrast, the item position is estimated by considering structured distances from respondents (Eq 2). The highly dependent structure of item response data differently embedded in the CBCL and YSR is first reflected in the item coefficient analysis. The high accordance exists in most item-wise coefficients between the CBCL and YSR, except for some outliers (Fig 2(a)). The items with a large discrepancy appear primarily related to sexual or physiological problems (Table 4). Parents do not respond positively to sexual and physiological issues of their children, even though their children are aware of these problems. This is predictable and is consistent with the findings in previous studies [50], which were revealed by using simple correlation analysis of the responses. In this respect, the current approach for analyzing item-wise coefficients in the latent space does not significantly differ from the direct comparison methods such as the Jaccard similarity measure (to compare the positive response count) in identifying discrepant items between the CBCL and YSR. However, the current study further characterizes the structured relationships between those items by presenting their positions in the latent space of the CBCL and YSR. As shown in Fig 2(b), those items are densely located on the upper boundary of the YSR, forming a clear cluster, but not in CBCL.

For items related to sexual or physiological problems with a large difference in item coefficients, *β*_*i*_, the positive response count is more endorsed in children than parents. Thus, those items have a more clustering tendency in the YSR. That means the respondent who positively responded to one of these outlier items also showed similar response patterns to other outlier items in the YSR data. In contrast, those patterns were not shown in the CBCL data. In other words, children think those items are highly related to each other, which is not considered the same for parents. This item dependency pattern was more evidenced in the item-item distance analysis.

In addition to providing a method to compare two scales using each item’s position, the advantage of the LSIRM-based approach is its ability to identify item-item distances in a structured way in the latent space. The item-item pairs that show differences between children and parents can be divided into two groups. One is the item pairs that children perceived as the underlying relationship while parents did not find them related to their children (Pairs 1–9 in Table 5). The other is item pairs that children did not find a relationship between domains in their behaviors, while parents observed a strong relationship between the two items in their children. For example, parents implicitly perceived the items in pair 1, i.e., “Act too young” and “Impulsive”, as not much relevance to children, while their children regarded the two as highly relevant. Furthermore, pairs 2–6 were related to “Trouble sleeping”. Trouble in sleeping may manifest in various syndromes, particularly in attention, such as inattentive or impulsive. However, it may be difficult for parents (as observers) to perceive the underlying connection. Meanwhile, parents consider that “Complains of loneliness” and “Stores up too many things he/she doesn’t need” are highly relevant items regarding their children, but children consider those items are irrelevant from their perspectives on themselves.

Note that the top item pairs showing the largest difference between parents and children in terms of item-item distance (Table 5) belong mostly to pairs between non-extreme syndromes (attention, social and thought problems (AP, SP, and TP)). Only a few item pairs from the same syndrome showed differences between children and parents, indicating that the item-item similarity within the same syndromes was relatively consistent across children and parents. This is conspicuous for items in syndromes with extreme properties (i.e., externalizing scores, internalizing scores), as was found in previous studies that report high consistency in extreme problem scores [7, 10, 13, 51].

Of note, the item-item pairs that showed distance differences between the children’s and parents’ views on the children (Fig 3(a)) are composed of items that do not belong to the list of items showing item-wise parameter differences between the CBCL and YSR (Fig 2). Meanwhile, the conventional response-based approach using the Jaccard similarity did not provide additional information compared to item-wise analysis of the difference between the CBCL and YSR. The item pairs with the most considerable difference of the Jaccard similarity (Fig 3(b)) are almost combinations of the outlier items in Fig 2(a). Therefore, the conventional dyadic comparison did not separate item-specific effects and the interactions within the CBCL or YSR.

Overlapped portion analysis of items’ latent positions between the CBCL and YSR also revealed similar results to the item-item distance analysis. Several item pairs in attention, social, and thought problems (AP, SP, and TP) have a significantly different relationship in the CBCL and YSR, especially for item pairs with one belonging to one of AP, SP, or TP and the other belonging to externalizing or internalizing scores Fig 4(c). In addition, most of the item pairs that showed high differences between children and parents were closer in the children compared to the parents (Fig 4).

Taking all results together, the current item-item dependency analysis using the LSIRM successfully specified the structural differences of children’s self-views and parents’ views on the children. Our latent space approach can reveal non-ignorable item-item dependence and identify that item-item dependence patterns and magnitude are fairly different between the CBCL and YSR data. In the CBCL-YSR context, where the test items indicate behavioral symptoms for certain syndromes, the identified difference in item dependency suggests that one could draw different conclusions, prevalent problems (or symptoms) and their relationships when data analysis was based on a single source, parent- or child- report only.

When we analyzed the latent position of the item syndrome, it shows directional order along the x-axis, i.e., the direction of ‘external’ to ‘internal’ axis (Fig 5(a)). This polarized property of externalizing and internalizing scores is consistent with the results of the item factor analysis [7]. Indeed, the axis of the fitted latent space does not have an interpretative meaning. However, by displaying the positions of item syndromes, one can make inferences about the space with reference to the position of each item syndrome. We found that the positions of syndromes in the YSR were generally closer than those in the CBCL. The congregation of the YSR syndromes reflects stronger interaction and a closer distance between syndromes in the YSR compared to the CBCL. This may be attributable to a tendency of positive answers in the YSR overall [51, 52]. The calculated cosine similarity (in Fig 5(b) and 5(c)) shows more apparent separation for items in externalizing and internalizing scores. Item syndromes belonging to externalizing or internalizing scores are generally perceived to be strongly related within the same group in both the CBCL and YSR, except for reduced association with SC (WD and SC) in the CBCL. However, for the attention, social, and thought problems (AP, SP, and TP), the perceived item relationship differs between the CBCL and YSR. This result is consistent with the findings based on the item-item distance pattern and their overlapped portion heatmap (Fig 4). Note that the distance among nonextreme syndromes (the attention, social, and thought problems) differ obviously between children and parents. Thus categorization of items done in the parent’s perspective may not be sufficient to characterize children’s diverse problems and demands a new attempt to make a more specific division of questionnaires.

Because the latent position of each respondent is mapped with the items’ latent position, clustering of respondents can identify not only a group of respondents who responded positively to the same item but also a group of respondents who responded positively to items with similar characteristics. This type of clustering index can be used to determine the relationship between each respondent or the discordance between parents and their children. For example, the clustering of respondents based on the distance between the item syndrome and each respondent (Fig 6(c) and 6(d)) shows that a cluster of the YSR located on the upper side of the latent space did not overlap with any specific symptoms and can be characterized by positive responses exclusively to items in Fig 2(b). This analysis revealed that the respondent-syndrome distances were not identical to some respondents in the CBCL and YSR spaces. The syndromes close to the respondent are the syndromes that the respondents (children in our context) are likely to have. Thus, this finding implies that one could draw different diagnostic conclusions about some children depending on whether children’s or parents’ reports were chosen for data analysis. These results also suggest that the conventional division of symptoms and clustering of individuals according to the dominant symptomatic problem may not necessarily apply to the division of the YSR.

Discrepancies between parent- and self-report measures have been commonly discussed, although examining and evaluating such discrepancies is an open area of research. The current study introduced a new and unique way of assessing parent- and self-report differences with a latent space approach using the LSIRM. Our empirical data analysis demonstrated that the two sets of measures from the two different informants did show much difference from the perspectives that have not been investigated with standard methods. Some of the differences that we identified have not been seen before and hold important practical implications. For example, behaviors and syndromes might show different structural relationships based on parent- or self-report measures. In addition, children might end up being diagnosed with a different set of behaviors and syndromes depending on whether parent- or self-report measure was used. Using the LSIRM, we mainly illustrated the potential discrepancies between parent- and child-report measures in terms of dependency. The identified CBCL-YSR differences from our proposed method illuminate and invite applied researchers to investigate and take a closer look at those discrepancies in other CBC-YSR datasets or other types of cross-informant measures.

In the current study, we did not examine how to resolve the identified differences, which is undoubtedly an important question, and we reserve it for future research. We primarily focused on the differences between informants in the latent space. Further analyses identifying the similarly responded items between informants, particularly items beyond internalizing and externalizing syndromes, remain as future research. Furthermore, the proposed model-based approach can be extended to a wide range of future work. First, our approach can be extended to non-binary item response data by choosing a suitable link function for non-binary item response data like in generalized linear models [20, 53]. Second, we can incorporate explanatory variables in our model to evaluate the discrepancy between the YSR and CBCL caused by the covariate information of the children, such as age [54], intelligence score, sex [1], culture, and ethnicity [11].

## Supporting information

S1 Appendix Metropolis-Hastings within Gibbs sampling for LSIRM. (PDF)

S2 Appendix Item divergence using KL-divergence. (PDF)

S1 Table Items’ syndrome membership. (PDF)

S2 Table Factor structures for CBCL and YSR. (PDF)
