# Supplementary material for: Analyzing differences between parent- and self-report measures with a latent space approach
Source: PLoS One. 2022 Jun 29;17(6):e0269376. doi: 10.1371/journal.pone.0269376 (PMC9242488; doi:10.1371/journal.pone.0269376)
Supplement: S1 Appendix — (PDF) [file pone.0269376.s001.pdf]

S1 Appendix

The likelihood part of the LSIRM is based on conditional independence, given the latent position of item and respondent. The data likelihood of item-response data with respondent $k = 1, .., N$ and item $i = 1, .., P$ can be defined as (Eq. 4). All model coefficient is estimated by the Bayesian approach so that the inference will be based on posterior samples. The priors of $\beta_i, \theta_k, \boldsymbol{w_i}$ and $\boldsymbol{z_k}$ are set to be multivariate normal distribution (MVN) with mean 0, and the priors for the variance parameters of random effect θ_k, $k = 1, .., N$ are set to be conjugate inverse-Gamma distribution.

$$\begin{aligned}
\beta_i|\tau_\beta^2 &\sim N(0, \tau_\beta^2), \tau_\beta^2 > 0 \\
\theta_k|\sigma^2 &\sim N(0, \sigma^2), \sigma^2 > 0 \\
\sigma^2 &\sim \text{Inv-Gamma}(a_\sigma, b_\sigma), a_\sigma > 0, b_\sigma > 0 \\
\boldsymbol{w_i} &\sim \text{MVN}_d(\boldsymbol{0}, \boldsymbol{I_d}) \\
\boldsymbol{z_k} &\sim \text{MVN}_d(\boldsymbol{0}, \boldsymbol{I_d}).
\end{aligned}$$

With this prior and data likelihood, the posterior kernel can be expressed as below.

$$\begin{aligned}
\pi(\beta, \theta, \sigma^2, Z, W|Y) \propto & P(Y|\beta, \theta, \sigma^2, Z, W)\pi(\beta, \theta, \sigma^2, Z, W) \\
\propto & \left[\prod_{k=1}^{N}\prod_{i=1}^{P} P(r_{k,i}|\beta_i, \theta_k, \boldsymbol{w_i}, \boldsymbol{z_k})\right] \\
& \left[\prod_{i=1}^{P}\pi(\beta_i)\prod_{k=1}^{N}\pi(\theta_j|\sigma^2)\pi(\sigma^2)\prod_{i=1}^{P}\pi(\boldsymbol{w_i})\prod_{k=1}^{P}\pi(\boldsymbol{z_k})\right].
\end{aligned}$$

This posterior kernel cannot be expressed with standard distribution, so the exact posterior density cannot be calculated and Gibbs sampler is used to sample each parameter sequentially from their conditional density.

All the Bayesian inferences are made through these posterior samples. For example, the posterior distribution of $\mathbb{P}(y_{ki} = 1)$ can be easily evaluated by the logistic formula

described above, with the posterior samples of $\beta_i, \theta_k, \boldsymbol{w_i}, \boldsymbol{z_k}$

$$
\begin{aligned}
\pi(\beta_i) &\propto \left[\prod_{k=1}^{N}\prod_{i=1}^{P} P(r_{k,i}|\beta_i, \theta_k, \boldsymbol{w_i}, \boldsymbol{z_k})\right] \times \left[N_{\beta_i}(0, \tau_\beta^2)\right] \\
\pi(\theta_k) &\propto \left[\prod_{k=1}^{N}\prod_{i=1}^{P} P(r_{k,i}|\beta_i, \theta_k, \boldsymbol{w_i}, \boldsymbol{z_k})\right] \times \left[N_{\theta_k}(0, \sigma^2)\right] \\
\pi(\boldsymbol{z_k}) &\propto \left[\prod_{k=1}^{N}\prod_{i=1}^{P} P(r_{k,i}|\beta_i, \theta_k, \boldsymbol{w_i}, \boldsymbol{z_k})\right] \times \left[\mathrm{MVN}_{d,\boldsymbol{z_k}}(\boldsymbol{0}, \boldsymbol{I_d})\right] \\
\pi(\boldsymbol{w_i}) &\propto \left[\prod_{k=1}^{N}\prod_{i=1}^{P} P(r_{k,i}|\beta_i, \theta_k, \boldsymbol{w_i}, \boldsymbol{z_k})\right] \times \left[\mathrm{MVN}_{d,\boldsymbol{w_i}}(\boldsymbol{0}, \boldsymbol{I_d})\right] \\
\pi(\sigma^2) &\propto \text{Inv-Gamma}\left(\left(\frac{N}{2} + a_\sigma\right), \frac{1}{2}\sum_{k=1}^{N}\theta_k^2 + b_\sigma\right).
\end{aligned}
$$

Specifically, because each conditional kernel may not be expressed as a standard distribution form, Metropolis-Hastings within Gibbs sampler is used. Furthermore, for the generalized case with missing data, the imputed value for non-respondent $(k \times i)$ pair can be sampled with the logistic formula at the start of each Gibbs sampler steps.
