# Supplementary material for: Analyzing differences between parent- and self-report measures with a latent space approach
Source: PLoS One. 2022 Jun 29;17(6):e0269376. doi: 10.1371/journal.pone.0269376 (PMC9242488; doi:10.1371/journal.pone.0269376)
Supplement: S2 Appendix — (PDF) [file pone.0269376.s002.pdf]

S2 Appendix

We utilized the Kullback–Leibler (KL) divergence that indicates the discrepancy between D_{ij}^{CBCL} and D_{ij}^{YSR} distributions, where $D_{ij}^{CBCL} = ||\boldsymbol{w_i} - \boldsymbol{w_j}||$ estimated in CBCL. Since KL-divergence is asymmetric measure and neither of CBCL and YSR can be treated as true distribution, we made the KL-divergence asymmetric matrix K which is defined as

$$K_{ij} = \begin{cases} KL(D_{ij}^{CBCL}||D_{ij}^{YSR}), & \text{if } i > j \\ 0, & \text{if } i = j \\ KL(D_{ij}^{YSR}||D_{ij}^{CBCL}), & \text{if } i < j. \end{cases}$$

to consider the discrepancy in both aspects. With K_{ij}, we can evaluate how distances between item pairs are different between CBCL and YSR, where distances represent dependency.

With KL-divergence asymmetric matrix K, the overall CBCL-YSR distances for each item can be measured with the following item-specific divergence index:

$$dii_i = \frac{\sum_{j \neq i} K_{ij}}{P - 1},$$

where $\sum_{j \neq i} K_{ij}$ indicates the row sum of K for item i (except for K_{ii}) and P is the dimension of K (i.e., the number of items). A large dii_i value indicates that the item's dependency with other test items was generally different between CBCL and the YSR.

We then examined the item-level divergence based on dii_i measures, an average KL-divergence for each item. Table 1 lists the top 10 items with the largest divergence differences between CBCL and YSR. Half of these items (Items 1, 10, 11, 78, 79) also appear in Table 5 for the top 10 largest differences in the item pair dependency, which makes sense. Those items are mainly located at the center of the YSR space while placed at the boundary in the CBCL space, meaning that YSR has more positive responses than CBCL. This is aligned with the reported tendency that YSR has more positive responses than CBCL in the literature.

Table 1. Items with the largest item-level divergence between the CBCL and YSR data. AP, SP and WD inside the brackets indicate the syndromes that these items belong to. [X] indicates that the item does not belong to any particular syndrome.

Questions
1. Acts too young for his/her age [AP]
77. Sleeps more than most kids during day and/or night [X]
10. Can't sit still, restless or hyperactive [AP]
78. Inattentive or easily distracted [AP]
11. Clings to adults or too dependent [SP]
79. Speech problem [SP]
42. Would rather be alone than with others [WD]
88. Sulks a lot [X]
49. Constipated, doesn't move bowels [X]
111. Withdrawn, doesn't get involved with others [WD]
