# Supplementary material for: Analyzing differences between parent- and self-report measures with a latent space approach
Source: PLoS One. 2022 Jun 29;17(6):e0269376. doi: 10.1371/journal.pone.0269376 (PMC9242488; doi:10.1371/journal.pone.0269376)
Supplement: S1 Table — (PDF) [file pone.0269376.s003.pdf]

S1 Table

Syndrome	Question
AB	3. Argues a lot
AB	16. Cruelty, bullying, or meanness to others
AB	19. Demands a lot of attention
AB	20. Destroys his/her own things
AB	21. Destroys things belonging to his/her family or others
AB	22. Disobedient at home
AB	23. Disobedient at school
AB	37. Gets in many fights
AB	57. Physically attacks people
AB	68. Screams a lot
AB	86. Stubborn, sullen, or irritable
AB	87. Sudden changes in mood or feelings
AB	89. Suspicious
AB	94. Teases a lot
AB	95. Temper tantrums or hot temper
AB	97. Threatens people
AB	104. Unusually loud
AD	14. Cries a lot
AD	29. Fears certain animals, situations, or places, other than school
AD	30. Fears going to school
AD	31. Fears he/she might think or do something bad
AD	32. Feels he/she has to be perfect
AD	33. Feels or complains that no one loves him/her
AD	35. Feels worthless or inferior
AD	45. Nervous, highstrung, or tense
AD	50. Too fearful or anxious

AD	52. Feels too guilty
AD	71. Self-conscious or easily embarrassed
AD	91. Talks about killing self
AD	112. Worries
AP	1. Acts too young for his/her age
AP	4. Fails to finish things he/she starts
AP	8. Can't concentrate, can't pay attention for long
AP	10. Can't sit still, restless or hyperactive
AP	13. Confused or seems to be in a fog
AP	17. Daydreams or gets lost in his/her thoughts
AP	41. Impulsive or acts without thinking
AP	61. Poor school work
AP	78. Inattentive or easily distracted
RBB	2. Drinks alcohol without parents' approval
RBB	26. Doesn't seem to feel guilty after misbehaving
RBB	28. Breaks rules at home, school, or elsewhere
RBB	39. Hangs around with others who get in trouble
RBB	43. Lying or cheating
RBB	63. Prefers being with older kids
RBB	67. Runs away from home
RBB	72. Sets fires
RBB	81. Steals at home
RBB	82. Steals outside the home
RBB	90. Swearing or obscene language
RBB	96. Thinks about sex too much
RBB	99. Smokes, chews, or sniffs tobacco
RBB	101. Truancy, skips school
RBB	105. Uses drugs for nonmedical purposes (don't include alcohol or tobacco)
SC	47. Nightmares
SC	51. Feels dizzy or lightheaded
SC	54. Overtired without good reason

SC	56A. Aches or pains (not stomach or headaches)
SC	56B. Headaches
SC	56C. Nausea, feels sick
SC	56D.A. Problems with eyes (not if corrected by glasses
SC	56E. Rashes or other skin problems
SC	56F. Stomachaches
SC	56G. Vomiting, throwing up
SP	11. Clings to adults or too dependent
SP	12. Complains of loneliness
SP	25. Doesn't get along well with other kids
SP	27. Easily jealous
SP	34. Feels others are out to get him/her
SP	36. Gets hurt a lot, accident-prone
SP	38. Gets teased a lot
SP	48. Not liked by other kids
SP	62. Poorly coordinated or clumsy
SP	64. Prefers being with younger kids
SP	79. Speech problem
TP	9. Can't get his/her mind off certain thoughts; obsessions
TP	18. Deliberately harms self or attempts suicide
TP	40. Hears sounds or voices that aren't there
TP	46. Nervous movements or twitching
TP	58. Picks nose, skin, or other parts of body
TP	66. Repeats certain acts over and over; compulsions
TP	70. Sees things that aren't there
TP	76. Sleeps less than most kids
TP	83. Stores up too many things he/she doesn't need
TP	84. Strange behavior
TP	85. Strange ideas
TP	100. Trouble sleeping
WD	5. There is very little he/she enjoys

WD	42. Would rather be along than with others
WD	65. Refuses to talk
WD	69. Secretive, keeps things to self
WD	75. Too shy or timid
WD	102. Underactive, slow moving, or lacks energy
WD	103. Unhappy, sad, or depressed
WD	111. Withdrawn, doesn't get inolved with others
.	6. Bowel movements outside toilet
.	7. Bragging, boasting
.	15. Cruel to animals
.	24. Doesn't eat well
.	44. Bites fingernails
.	49. Constipated, doesn't move bowels
.	53. Overeating
.	55. Overweight
.	59. Plays with own sex parts in public
.	60. Plays with own sex parts too much
.	73. Sexual problems
.	74. Showing off or clowning
.	77. Sleeps more than most kids during day and/or night
.	80. Stares blankly
.	88. Sulks a lot
.	92. Talks or walks in sleep
.	93. Talks too much
.	98. Thumb-sucking
.	106. Vandalism
.	107. Wets self during the day
.	108. Wets the bed
.	109. Whining
.	110. Wishes to be of opposite sex

Table 1. Item's syndrome membership, the items without syndrome are denoted as '.'
