# Supplementary material for: Analyzing differences between parent- and self-report measures with a latent space approach
Source: PLoS One. 2022 Jun 29;17(6):e0269376. doi: 10.1371/journal.pone.0269376 (PMC9242488; doi:10.1371/journal.pone.0269376)
Supplement: S2 Table — (PDF) [file pone.0269376.s004.pdf]

S2 Table

	CBCL				YSR			
	F1	F2	F3	F4	F1	F2	F3	F4
Q01			-.439					.356
Q02		.748			.309	.505	.304	
Q03				.762		.510		
Q04			-.464					
Q05	-.488				.426			
Q06				.376				
Q07				.475		.359		
Q08			-.828			.323		.314
Q09	-.342		-.313		.316		-.315	
Q10			-.381	.415			-.328	
Q11	-.344	-.329						
Q12	-.447				.677			
Q13	-.318		-.601		.548			
Q14	-.562				.495			
Q15			-.365		.353			
Q16				.718		.613		
Q17			-.571		.309			
Q18	-.443	.301			.447			
Q19				.756				
Q20				.509		.360		
Q21				.594		.509	-.319	
Q22				.699		.708		
Q23				.627		.794		
Q24					.352			
Q25				.544			-.309	

Q26				.622		.380	
Q27				.607	.556		
Q28		.395		.623		.785	
Q29	-.440						
Q30	-.498				.557		
Q31	-.397				.454		
Q32	-.414				.498		
Q33	-.385			.565	.538		
Q34				.616	.389		
Q35	-.524				.740		
Q36	-.345						-.362
Q37				.638		.502	-.415
Q38				.388			-.381
Q39		.355		.388		.548	
Q40	-.404						-.696
Q41			-.430	.505		.358	
Q42	-.472				.433		
Q43		.403		.372		.563	
Q44							
Q45	-.526				.750		
Q46					.359		-.373
Q47	-.334						-.322
Q48				.507		.308	-.370
Q49	-.373						
Q50	-.714				.839		
Q51	-.568				.417		
Q52	-.651				.624		
Q53							
Q54	-.444				.463		
Q55							
Q56A	-.348						-.456

Q56B	-.396						-.522	
Q56C	-.594						-.487	
Q56D							-.367	
Q56E	-.319						-.418	
Q56F	-.480						-.525	
Q56G	-.357						-.667	
Q57				.617		.546	-.359	
Q58			-.313					
Q59			-.869					.590
Q60			-.621					.428
Q61		.327	-.314			.414		
Q62			-.423		.339			
Q63						.316		
Q64			-.422					
Q65	-.358						-.353	
Q66			-.395				-.306	
Q67		.463		.356		.467		
Q68				.599				
Q69	-.393	.424			.400			
Q70	-.368		-.360				-.656	
Q71	-.484				.669			
Q72		.313	-.443	.325		.584		
Q73		.323						
Q74			-.344	.489		.435		.451
Q75	-.503				.511			
Q76								
Q77	-.401	.355						
Q78			-.798					.370
Q79			-.448				-.356	
Q80			-.459					.369
Q81		.400				.551		

Q82		.518	-.370			.732		
Q83								
Q84			-.341				-.316	
Q85			-.369		.366			
Q86				.520		.363		
Q87	-.470			.396	.377			
Q88	-.470			.407				.610
Q89				.481				
Q90				.458		.568		
Q91	-.350			.530	.576			
Q92								.527
Q93		-.344		.609				.426
Q94				.649		.663		
Q95				.687		.442		
Q96			-.337	.346		.471		
Q97				.750		.701		
Q98	.334			.310				.511
Q99		.831				.749		
Q100	-.301				.357		-.353	
Q101		.681				.563		
Q102	-.497				.560			
Q103	-.680				.720			
Q104				.573			-.345	.427
Q105		.862				.746		
Q106		.374	-.335	.392				.525
Q107			-.408					.762
Q108								.393
Q109				.554				.720
Q110	-.552				.427			
Q111	-.530							
Q112	-.550				.826			

Table 1. Factor structures for CBCL and YSR. With both datasets, 4-factor solutions were optimal with chi-squared goodness-of-fit 54121.22 (63613.37) with clearly significant p-value, RMSEA 0.037 (0.031) and CFI 0.949 (0.952) in CBCL (YSR). Loadings smaller than 0.3 are suppressed. For the first 2 factors, two data shows similar patterns: the first large factor covers most items of the AD syndrome (an internalizing syndrome), and the second large factor covers most items of the RBB syndrome, an externalizing syndrome. However, the third factor loaded on AP, SP, and TP syndromes in CBCL, while in YSR it is loaded on SC and WD syndromes. The fourth factor is loaded on the items with no syndrome membership in YSR, while in CBCL this factor is loaded on most of the AB syndrome.
